# Hypertension guidelines and coronary artery calcification among South Asians: Results from MASALA and MESA

**DOI:** 10.1016/j.ajpc.2021.100158

**Published:** 2021-02-12

**Authors:** Jaideep Patel, Anurag Mehta, Mahmoud Al Rifai, Michael J Blaha, Khurram Nasir, John W McEvoy, Ambarish Pandey, Alka M Kanaya, Namratha R Kandula, Salim S Virani, Antonio Abbate, Gregory Hundley, Laurence Sperling, Parag H Joshi

**Affiliations:** a Pauley Heart Center, VCU Medical Center, United States; b Ciccarone Center for the Prevention of Cardiovascular Disease, Baltimore, United States; c Emory Clinical Cardiovascular Research Institute, Division of Cardiology, Department of Medicine, Emory University School of Medicine, Georgia, United States; d Section of Cardiology, Department of Medicine, Baylor College of Medicine, United States; e Division of Cardiology, Houston Methodist Hospital, United States; f National Institute for Prevention and Cardiovascular Health, National University of Ireland, Galway, Ireland; g Division of Cardiology, University of Texas Southwestern Medical Center, 5323 Harry Hines Boulevard, E5.730F, Dallas, TX 75390, United States; h Department of Medicine, University of California, San Francisco, United States; i Department of Internal Medicine, Northwestern University, United States; j Section of Cardiology, Michael E. DeBakey Veterans Affairs Medical Center, United States

**Keywords:** South Asian, Prevention, Hypertension, Risk, Ethnic, ASCVD

## Abstract

Untreated hypertension may contribute to increased atherosclerotic cardiovascular disease (ASCVD) risk in South Asians (SA). We assessed HTN prevalence among untreated adults free of baseline ASCVD from the MASALA & MESA studies. The proportion of participants who received discordant recommendations regarding antihypertensive pharmacotherapy use by the 2017-ACC/AHA and JNC7 Guidelines across CAC score categories in each race/ethnic group was calculated. Compared with untreated MESA participants (*n* = 3896), untreated SA (*n* = 445) were younger (55±8 versus 59±10 years), had higher DBP (73±10 versus 70±10 mmHg), total cholesterol (199±34 versus 196±34 mg/dL), statin use (16% versus 9%) and CAC=0 prevalence (69% versus 58%), with fewer current smokers (3% versus 15%) and lower 10-year-ASCVD-risk (6.4% versus 9.9%) (all *p*<0.001). A higher proportion of untreated MASALA and MESA participants were diagnosed with hypertension and recommended anti-hypertensive pharmacotherapy according to the ACC/AHA guideline compared to JNC7 (all *p*<0.001). Overall, discordant BP treatment recommendations were observed in 9% SA, 11% Whites, 15% Blacks, 10% Hispanics, and 9% Chinese-American. In each race/ethnic group, the proportion of participants receiving discordant recommendation increased across CAC groups (all *p*<0.05), however was highest among SA (40% of participants). Similar to other race/ethnicities, a higher proportion of SA are recommended anti-hypertensive pharmacotherapy by ACC/AHA as compared with JNC7 guidelines. The increase was higher among those with CAC>100 and thus may be better at informing hypertension management in American South Asians.

Untreated hypertension may contribute to the increased atherosclerotic cardiovascular disease (ASCVD) risk seen in South Asians (SA) [1,2]. We sought to estimate the change in prevalence of untreated hypertension among SA per recent and prior hypertension guidelines [2,3]. We also studied the association of coronary artery calcium (CAC) score with guideline-recommended blood-pressure (BP) categories among SA and compared with four other race/ethnic groups.

We included asymptomatic participants from two community-based cohorts: MASALA (Mediators of Atherosclerosis in South Asians Living in America) and MESA (Multi-Ethnic Study of Atherosclerosis) [4,5]. Briefly, MESA is a multi-ethnic, community-based, prospective cohort study of 6814 men and women aged 45–84 years, free from baseline clinical ASCVD. Participants were enrolled between July 2000 and September 2002 at 6 field centers in the US (Baltimore, Maryland; Chicago, Illinois; Forsyth County, North Carolina; Los Angeles County, California; New York, New York; and St. Paul, Minnesota) and identified themselves as Non-Hispanic White, African American, Hispanic, or Chinese American [5]. MASALA is a community-based prospective cohort study of 906 asymptomatic adults, aged 40–79 years old of SA ancestry, free of known clinical ASCVD. Participants were enrolled between October 2010 and March 2013, from 2 clinical sites (University of California, San Francisco and Northwestern University [4]. The study was approved by the institutional review boards at each center. All MASALA and MESA participants provided a written informed consent, respectively [4,5]. Per MASALA data use guidelines requiring IRB approval at an author's home institution, IRB approval was obtained and deemed exempt from review by the Emory University IRB. Baseline data for MASALA and MESA were collected between 2010 and 2013 and 2000–2002, respectively [4]. BP categories at which pharmacotherapy is recommended was defined according to the 2017-AHA/ACC guideline [Stage1: systolic blood-pressure (SBP) 130–139 or diastolic blood-pressure (DBP) 80–89 mmHg *and* clinical-ASCVD or 10-year-ASCVD-risk≥10%, Stage2: BP≥140/90 mmHg] and the Seventh Report of the Joint National Committee on Treatment of High Blood-Pressure (JNC7) (BP≥140/90 mmHg) [2,3]. Details of the CAC quantification methods implemented in each of the two studies have been reported previously [4,5]. In MESA, CAC was measured using either an electron-beam (EB) or multi-detector CT. All images were interpreted at the Los Angeles Biomedical Research Center (Torrance, CA). The intraobserver and interobserver agreement for CAC were excellent (kappa statistics, 0.93 and 0.90, respectively). In MASALA, CAC was assessed using a cardiac-gated EBCT scanner and all images were analyzed at the Los Angeles Biomedical Research Center according to MESA study methods [4]. In both studies CAC scans were interpreted blinded to race/ethnicity and quantified using the Agatston scoring system [5]. Both intraobserver and interobserver agreement for CAC are expected to be similar for MASALA given identical scanning protocols and imaging interpretation center.

To align with MESA, we excluded MASALA participants aged <45-years (*n* = 126). We excluded participants on baseline anti-hypertensive pharmacotherapy (MASALA/MESA, *n* = 277/*n* = 2267). We excluded participants with diabetes (DM; *n* = 57/*n* = 364) or chronic kidney disease (CKD; *n* = 4/*n* = 298), since both prior and current guidelines recommend similar approaches for these conditions. A considerable proportion of untreated participants with DM (54%/42%) and CKD (25%/53%) would have qualified for anti-hypertensive pharmacotherapy by both guidelines (BP≥130/80 mmHg). The untreated population consisted of 445 MASALA and 3896 MESA (1672 White, 854 Black, 884 Hispanic, and 486 Chinese-American) participants. The 10-year-ASCVD-risk was estimated using the pooled cohort equations; SA and Chinese-Americans were categorized as “other” in the PCE. The proportion of participants receiving discordant recommendations regarding anti-hypertensive pharmacotherapy use (no treatment by JNC7 [BP 120–139/80–89] and treatment by ACC/AHA [BP 130–139/80–89 with 10-year ASCVD risk >10%]) was calculated and stratified by CAC score categories (=0, 1–100, >100) in each race/ethnic group.

Compared with MESA participants, SA were younger (55±8 versus 59±10 years), had higher DBP (73±10 versus 70±10 mmHg), total cholesterol (199±34 versus 196±34 mg/dL), statin use (16% versus 9%) and CAC=0 prevalence (69% versus 58%), with fewer current smokers (3% versus 15%) and lower 10-year-ASCVD-risk (6.4% versus 9.9%) (all Mann-Whitney U [for continuous variables] and chi-square [for categorical variables] *p*<0.001). The prevalence of untreated hypertension across race/ethnicity is shown in Fig. 1. A higher proportion of untreated MASALA and MESA participants were diagnosed with hypertension and recommended anti-hypertensive pharmacotherapy according to the ACC/AHA guideline compared to JNC7 (all chi-square *p*<0.001). Among SA, more than half qualified for lifestyle modifications and 17% were recommended anti-hypertensive pharmacotherapy by ACC/AHA compared to 8% by JNC7 (chi-square *p*<0.001). Overall, discordant BP treatment recommendations were observed in 9% SA, 11% Whites, 15% Blacks, 10% Hispanics, and 9% Chinese-Americans. In each race/ethnic group, the proportion of participants receiving discordant recommendation increased across CAC categories (all chi-square *p*<0.05, Fig. 2). This trend aligned with increasing ASCVD risk across CAC categories in each race/ethnic group (all p-trend<0.001).

**Fig. 1:**
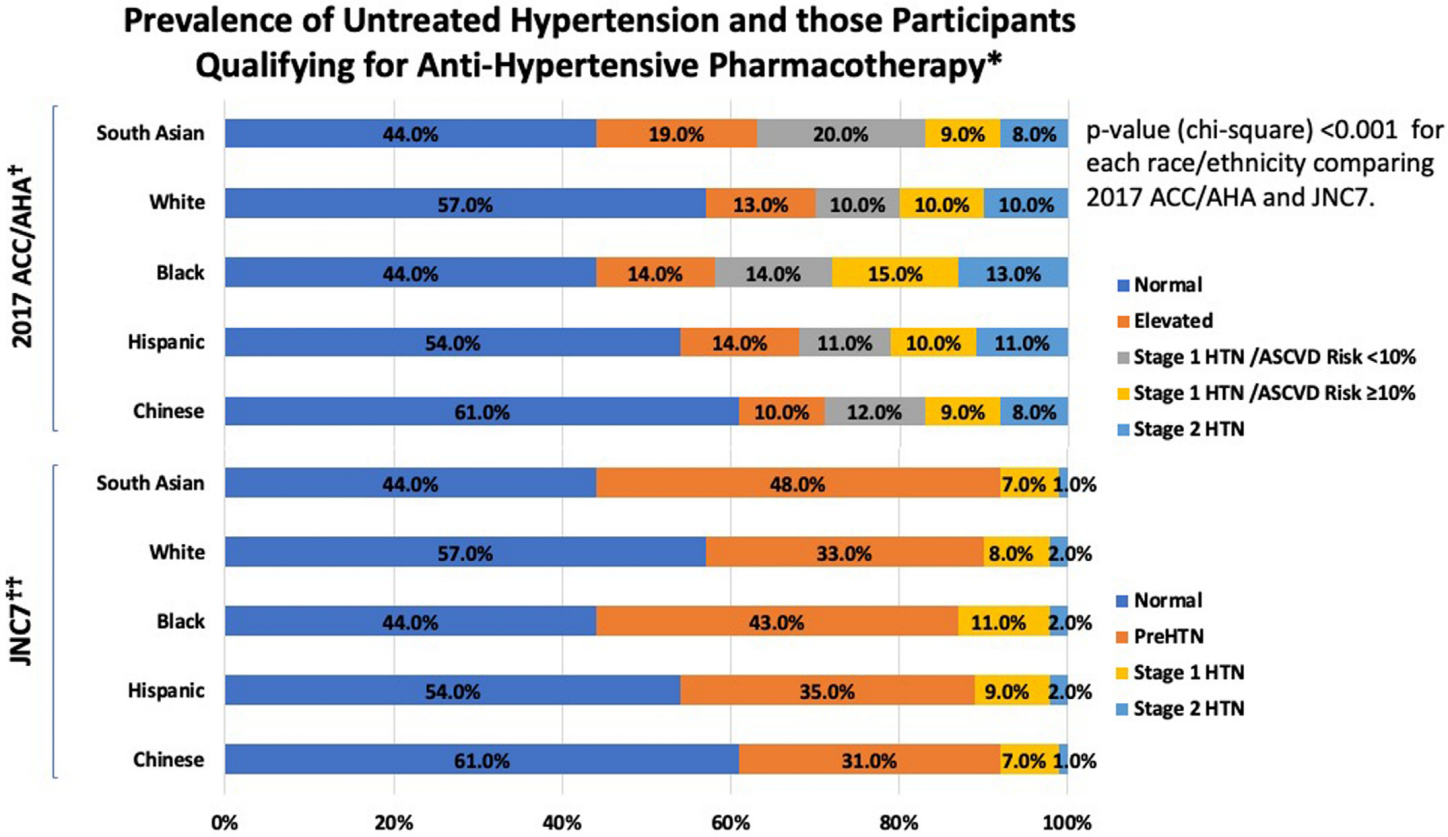
Prevalence of Untreated Hypertension and those Participants Qualifying for Anti-Hypertensive Pharmacothearpy.* *Excludes those with diabetes mellitus or chronic kidney disease as treatment recommendations are similar. □ *p*<0.001 for South Asians, *p*<0.001 for Blacks, *p* = 0.573 for Hispanics, and *p* = 0.118 for Chinese-Americans (White as reference) for comparison of percentages across each race/ethnicity group □□ *p*<0.001 South Asians, *p*<0.001 for Blacks, *p* = 0.606 for Hispanics, and *p* = 0.430 for Chinese-Americans for (White as reference) for comparison of percentages across each race/ethnicity group. ACC/AHA – American College of Cardiology/American Heart Association; ASCVD Risk – atherosclerotic cardiovascular disease risk calculated by the Pooled Cohorts Equations; HTN – hypertension; JNC7 - Seventh Report of the Joint National Committee on Treatment of High Blood-Pressure. Blood Pressure Definitions: *2017 ACC/AHA*: Normal: systolic blood pressure (SBP) <120 mmHg and diastolic blood pressure (DBP) <80 mmHg; Elevated Blood Pressure: SBP 120–129 mmHg and DBP <80 mmHg; Stage 1: SBP 130–139 or DBP 80–89 mmHg; Stage 2: SBP ≥140 mmHg or DBP ≥ 90mmHg J*NC7*: Normal: SBP <120 and DBP <80; PreHTN: SBP 120–139 mmHg or DBP 80–89 mmHg; Stage 1: SBP 140–159 or DBP 90–99 mmHg; Stage 2: SBP ≥160 mmHg or DBP ≥100 mmHg.

**Fig. 2:**
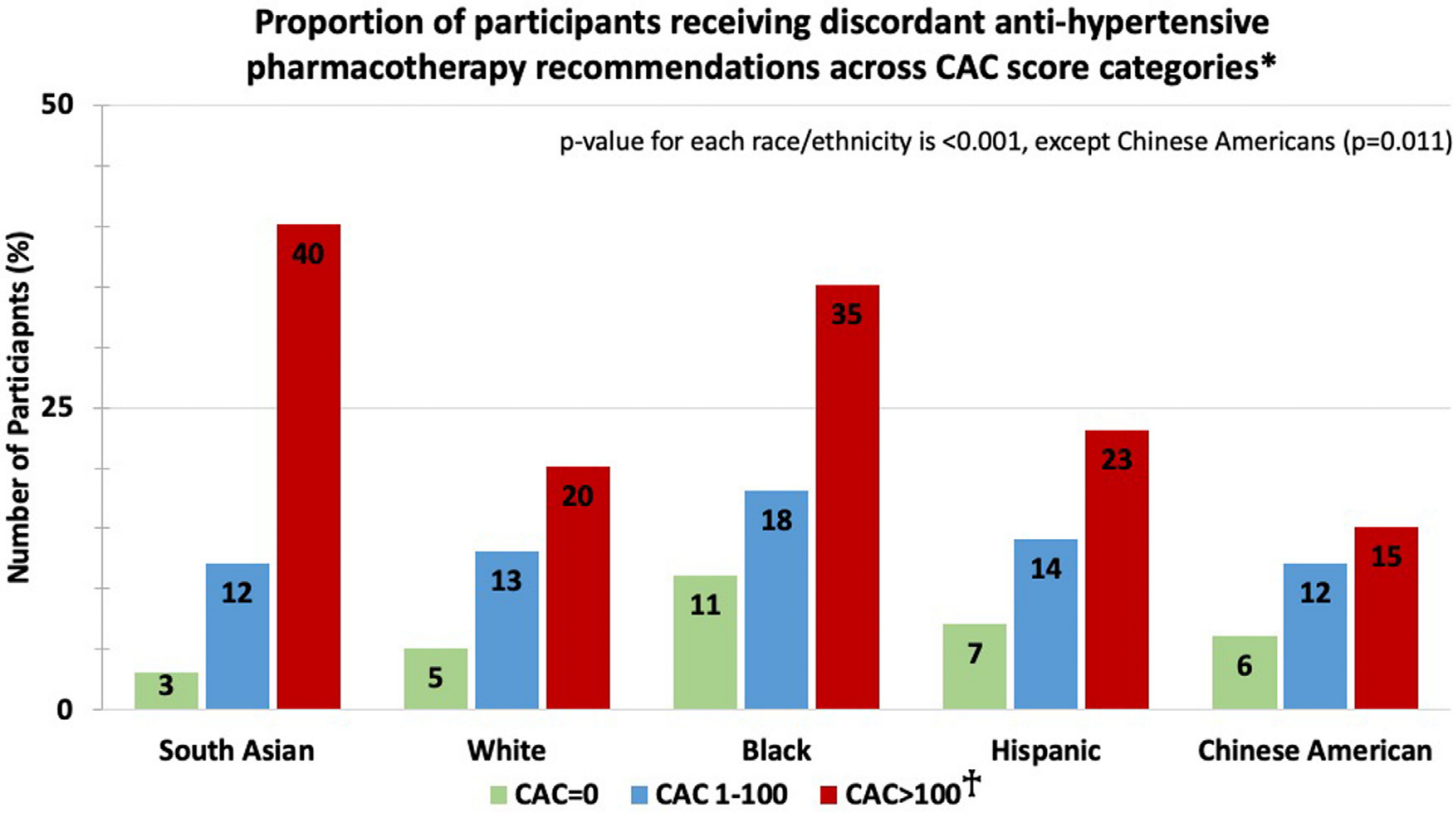
Proportion of participants receiving discordant anti-hypertensive pharmacotherapy recommendations across CAC score categories*. *Discordant recommendations: Prehypertension by JNC7 and Stage 1 hypertension with 10-year ASCVD risk ≥10% by ACC/AHA guideline. □p-value for discordant recommendation in each CAC category across each race/ethnic group (Reference group is White race) CAC=0: *p* = 0.280 for South Asians, *p*<0.001 for Blacks, *p* = 0.079 for Hispanics, and *p* = 0.469 for Chinese-Americans CAC 1–100: *p* = 0.712 for South Asians, *p* = 0.150 for Blacks, *p* = 0.939 for Hispanics, and *p* = 0.842 for Chinese-Americans CAC>100: *p* = 0.003 for South Asians, *p* = 0.003 for Blacks, *p* = 0.528 for Hispanics, and *p* = 0.309 for Chinese-Americans CAC – coronary artery calcium.

The majority of SA qualified for interventions aimed at lifestyle optimization, providing an opportunity to address modifiable cardiometabolic risk-factors [1, 6]. Additionally, the proportion of participants that would qualify for anti-hypertensive pharmacotherapy per ACC/AHA but not by JNC7 guidelines was higher among those with CAC >100, across all race/ethnicities. Previous studies have demonstrated that CAC burden is closely aligned with ASCVD risk estimated using the PCE. It is likely that the higher discordance in recommendations among participants with CAC>100 is driven by the ACC/AHA guideline-endorsed inclusion of ASCVD risk in determining anti-hypertensive pharmacotherapy use.

Further investigation should focus on implementing evidence-based hypertension guidelines and achieving optimal engagement in lifestyle changes and medication adherence in SA. Higher DBP in MASALA should be explored as a potential contributor to the increased risk of ASCVD in SA [2]. Future data on incident ASCVD events in MASALA will allow for validation of the association between BP and CAC.

Strengths of the study include the high quality assessment of risk markers, such as CAC, and standardized measurement of multiple risk factors. MASALA and MESA include a substantial proportion of previously understudied ethnic groups, derived from various geographical locations in the US [4,5]. Lastly, both study populations are primary prevention cohorts in whom ASCVD risk estimation is used to guide the initiation of preventive pharmacotherapies [6]. A limitation of this study is that BP was obtained at a single visit, whereas guidelines suggest averaging BP measurements over ≥2 visits. Lastly, MASALA enrolled participants 10-years after MESA and may be subject to secular temporal trends in BP management.

In conclusion, similar to other race/ethnicities, a higher proportion of SA are recommended anti-hypertensive pharmacotherapy by ACC/AHA as compared with JNC7 guidelines. This increase was higher among those with greater subclinical atherosclerosis burden and thus, may be better at informing hypertension management in SA living in America.

## Disclosures

Dr. Nasir has reported consulting for Regeneron; and is on the advisory board of Quest Diagnostics. Dr. Joshi reports grant support from AHA, NovoNordisk, NASA; Consulting from Regeneron and Bayer, Equity in G3 Therapeutics. Dr Virani reports research support from the Department of Veterans Affairs, World Heart Federation, and Tahir and Jooma Family; Honorarium from the American College of Cardiology (Associate Editor for Innovations, acc.org); Steering Committee Member for the Patient and Provider Assessment of Lipid Management (PALM) registry at the Duke Clinical Research Institute (no financial remuneration). All other authors have reported that they have no relationships relevant to the contents of this paper to disclose.

## Author contributions

None of the authors received any funding any funding for the preparation of this manuscript. JP conceived the study design and wrote the initial draft of the manuscript; AM performed the analytic calculations and wrote the initial draft of the manuscript; MAR, MJB, KN, JWM, AP, AK, NRK, SSV, AA, GH, LS, and PHJ contributed to the design, writing, and provided critical review of the manuscript

## Declaration of Competing Interest

The authors declare that they have no known competing financial interests or personal relationships that could have appeared to influence the work reported in this paper.

## Appendix Supplementary materials

Image, application 1
